# 1015. Real World Use of Dalbavancin and Oritavancin for Optimized Outpatient Antimicrobial Therapy in Patients Inappropriate for Standard Therapy

**DOI:** 10.1093/ofid/ofac492.856

**Published:** 2022-12-15

**Authors:** Brittani Weichman, Amanda Bushman, Angela Aarhus, David A Terrero Salcedo, Ty C Drake

**Affiliations:** UnityPoint Health Meriter, Madison, Wisconsin; UnityPoint Health Des Moines, Urbandale, Iowa; UnityPoint Health Des Moines, Urbandale, Iowa; UNITYPOINT CLINIC, DES MOINES, Iowa; Houston Methodist Willowbrook Hospital, Houston, Texas

## Abstract

**Background:**

Dalbavancin and oritavancin are long acting lipoglycopeptide antibiotics (LaLGP) approved for treatment of acute bacterial skin and skin structure infections (ABSSSI) caused by susceptible gram-positive organisms. These antibiotics have shown promise in treatment of non-FDA approved gram-positive infections including bacteremia, endocarditis, pneumonia, and osteomyelitis. The aim of this review was to determine indication, length of therapy, treatment failures, and adverse drug events (ADE) in patients receiving LaLGP at our institution.

**Methods:**

Retrospective study of adult patients that received LaLGP within an integrated health system consisting of 3 hospitals and 2 outpatient infusion centers, between July 2016 and February 2022. Data collection included dosing regimen specifics for both LaLGP and non-LaLGP, indication and justification for LaLGP use, culture results, labs, ADE, incidence of readmission and Emergency (ED)/Urgent Care presentation, and treatment failure defined as readmission or return to the ED/Urgent care within 90 days of therapy completion.

**Results:**

36 patients received LaLGP during the study period. A majority of patients received LaLGP for an indication other than ABSSSI (64%)(Table 1). Four patients were lost to follow-up but included in analysis of indication and justification for LaLGP use. *Staphylococcus aureus* (30% MRSA, 26% MSSA) was the most commonly identified pathogen in patients who received LaLGP for non-ABSSSI indications.

Justification for use of LaLGP over standard therapy is shown in Table 2. The median duration of standard therapy prior to transitioning to LaLGP was 7 days.

Table 3 shows agent selection, number of doses, and outcomes for ABSSSI indications vs non-ABSSSI indications. ABSSSI patients received a maximum of 2 doses; non-ABSSSI indications received 1-8 doses. Re-admission rates were higher in the ABSSSI group compared to non-ABSSSI while ED/Urgent care presentations were higher in non-ABSSSI indications.

**Figure:**
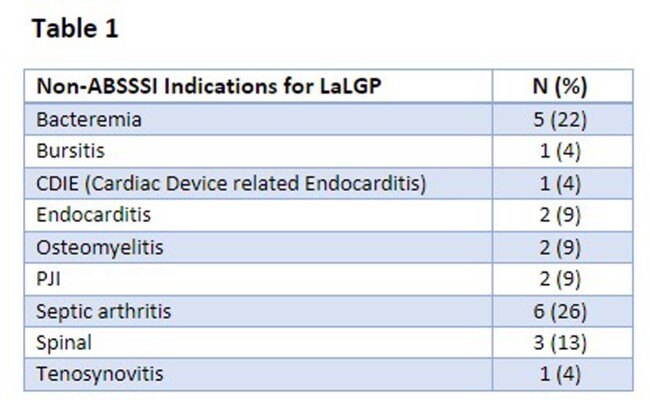

**Figure:**
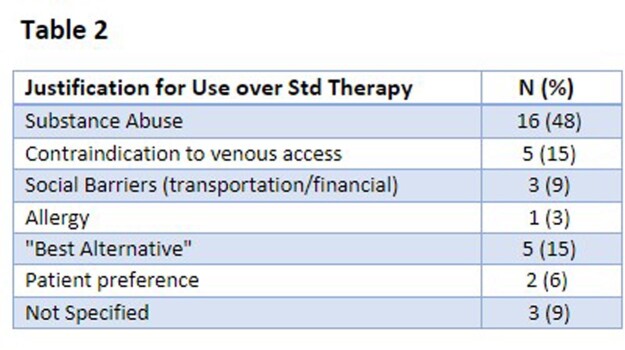

**Figure:**
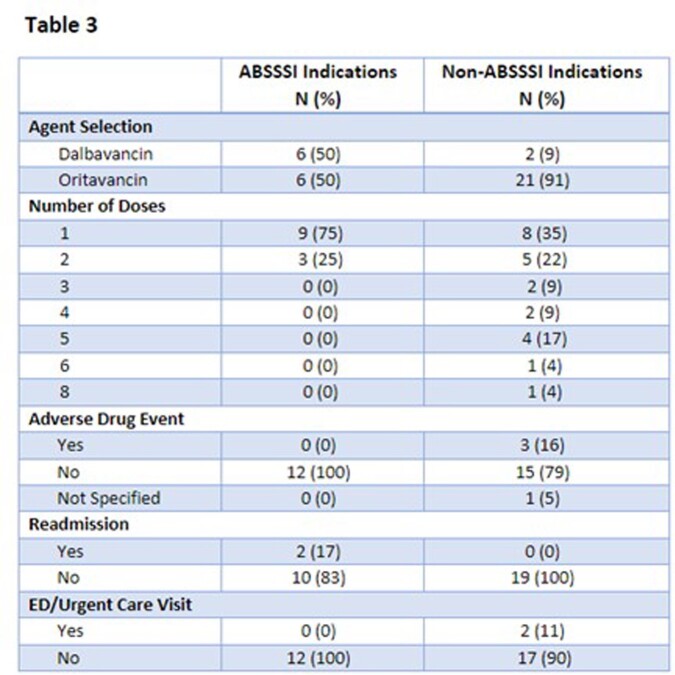

**Conclusion:**

LaLGP is a viable option for therapy completion after improvement is seen while on standard therapy in patients where daily outpatient antimicrobial therapy is not feasible. While LaLGP is currently only approved for ABSSSI, they appear to be safe and effective for other indications.

**Disclosures:**

**All Authors**: No reported disclosures.

